# Patterns of Genetic Variability in Island Populations of the Cane Toad (*Rhinella marina*) from the Mouth of the Amazon

**DOI:** 10.1371/journal.pone.0152492

**Published:** 2016-04-13

**Authors:** Adam Rick Bessa-Silva, Marcelo Vallinoto, Davidson Sodré, Divino Bruno da Cunha, Dante Hadad, Nils Edvin Asp, Iracilda Sampaio, Horacio Schneider, Fernando Sequeira

**Affiliations:** 1 Laboratório de Evolução (LEVO), Instituto de Estudos Costeiros (IECOS), Universidade Federal do Pará, Campus de Bragança, Pará, Brasil; 2 CIBIO-InBIO, Centro de Investigação em Biodiversidade e Recursos Genéticos, Laboratório Associado, Campus Agrário de Vairão, Universidade do Porto, Vairão, Portugal; 3 Laboratório de Geologia Costeira (LAGECO), Instituto de Estudos Costeiros (IECOS), Universidade Federal do Pará, Campus de Bragança, Pará, Brasil; 4 Laboratório de Filogenômica e Bioinformática, Instituto de Estudos Costeiros (IECOS), Universidade Federal do Pará, Campus de Bragança, Pará, Brasil; Fordham University, UNITED STATES

## Abstract

The Amazonian coast has several unique geological characteristics resulting from the interaction between drainage pattern of the Amazon River and the Atlantic Ocean. It is one of the most extensive and sedimentologically dynamic regions of the world, with a large number of continental islands mostly formed less than 10,000 years ago. The natural distribution of the cane toad (*Rhinella marina*), one of the world’s most successful invasive species, in this complex Amazonian system provides an intriguing model for the investigation of the effects of isolation or the combined effects of isolation and habitat dynamic changes on patterns of genetic variability and population differentiation. We used nine fast-evolving microsatellite loci to contrast patterns of genetic variability in six coastal (three mainlands and three islands) populations of the cane toad near the mouth of the Amazon River. Results from Bayesian multilocus clustering approach and Discriminant Analyses of Principal Component were congruent in showing that each island population was genetically differentiated from the mainland populations. All *F*_*ST*_ values obtained from all pairwise comparisons were significant, ranging from 0.048 to 0.186. Estimates of both recent and historical gene flow were not significantly different from zero across all population pairs, except the two mainland populations inhabiting continuous habitats. Patterns of population differentiation, with a high level of population substructure and absence/restricted gene flow, suggested that island populations of *R*. *marina* are likely isolated since the Holocene sea-level rise. However, considering the similar levels of genetic variability found in both island and mainland populations, it is reliable to assume that they were also isolated for longer periods. Given the genetic uniqueness of each cane toad population, together with the high natural vulnerability of the coastal regions and intense human pressures, we suggest that these populations should be treated as discrete units for conservation management purposes.

## Introduction

**Fig 1:**
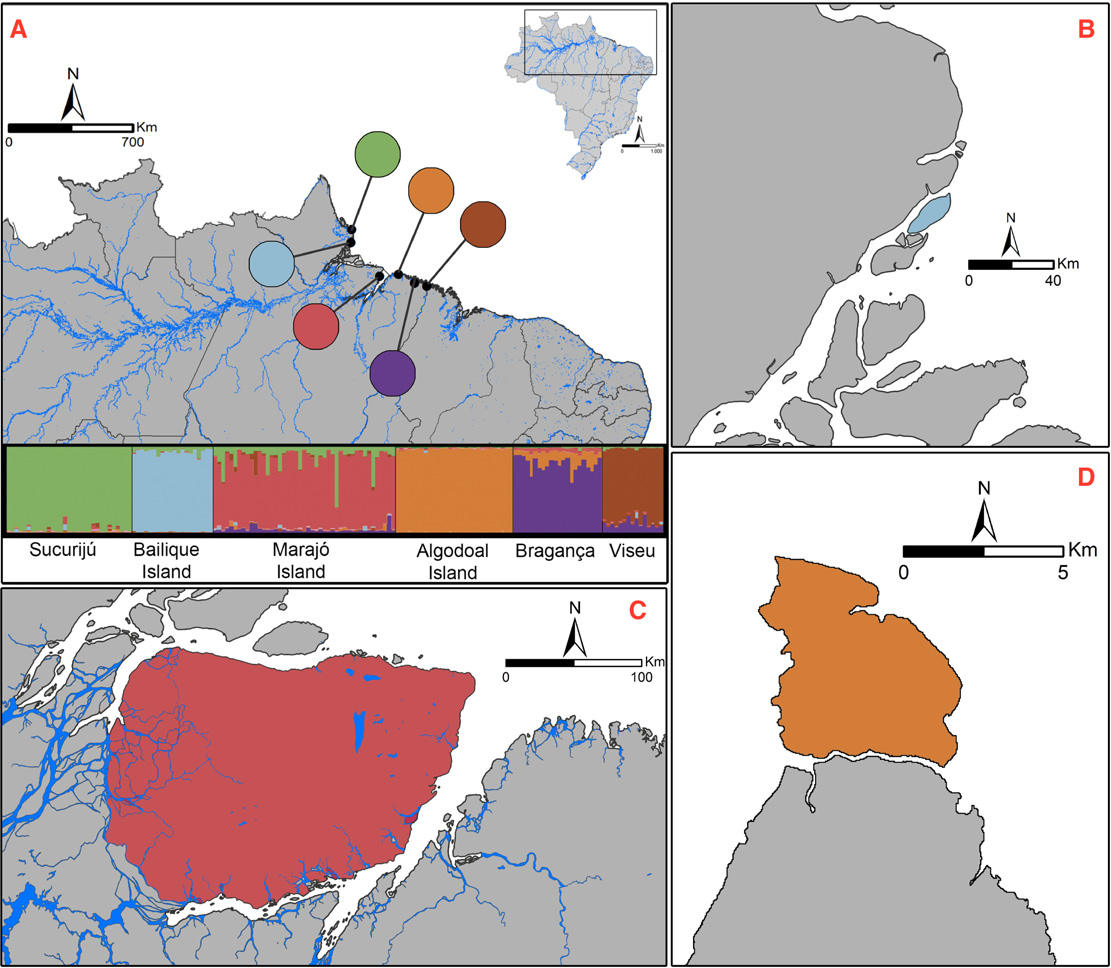
Map of sampled localities of *Rhinella marina* and Bayesian clustering results of STRUCTURE analysis for microsatellites (*K* = 6; see results). (A) Each individual is represented as a vertical line partitioned into *K* colored segments, whose length is proportional to the individual’s estimated membership coefficient. A black line separates individuals of different populations. These are labeled below the figure and are sorted from north to south (from the left to the right of the figure). (B) Bailique Island in blue; (C) Marajó Island in red; and (D) Algodoal Island in orange. Map created using ArcGIS 10.2.

The Amazonian coastline is a highly dynamic natural system that changes continuously in response to a variety of phenomena that differ considerably in timing and duration, such as Holocene sea level rises, tectonic activities and hydrologic dynamics, including drainage, sedimentation and erosion patterns [1, 2]. Indeed, the Amazonian coast (Fig 1), comprising approximately 1,200 km from the Brazilian state of Amapá, in the north, to Maranhão, in the east [3], is characterized by a large number of continental islands formed by consequence of the distinct geomorphological processes [1, 4, 5]. In particular, the sea-level rise between late Pleistocene and Middle Holocene is generally considered to be the main event that originated the continental islands by separation from the adjacent mainland [6–8]. These continental islands, comparative to oceanic ones, tend to be younger and to present distinct evolutionary patterns due to their formation from vicariance, rather than colonization or founding event in novel environmental conditions such as in oceanic islands. Even so, the effects of island formation and the variability in population genetics in these region are still only poorly investigated.

In small populations, genetic drift can lead to a random loss of alleles, and inbreeding may then occur with consequent survival and fitness reductions, an increase of the frequency of deleterious mutations and susceptibility to emerging pathogens [9, 10]. For example, among amphibians, there are a vast number of studies reporting a correlation between physical abnormalities and low genetic diversity and increased genetic inbreeding in small isolated populations [11–16]. Island populations are often small and isolated with reduced effective population size, being thus more vulnerable to stochastic genetic processes and environmental changes [17, 18].

The islands in the Amazon River vary considerably in the level of man-induced habitat loss and/or degradation. While in some islands the main cause of environmental disturbance is pasture and agriculture, others suffered from the direct or indirect influence of harsh chemicals and mineral ores exploitation [19–23]. In this sense, the natural occurrence of the cane toad (*Rhinella marina*) across the complex system of Amazonian coastal islands is an interesting case study for the investigation of the effects of isolation and/or combined effects of isolation and habitat dynamic changes on patterns of genetic variability and population differentiation. The cane toad is a widely distributed species throughout Central and South America [24, 25], and is well known as a successful exotic invaders, especially in Australia, where its rapid spread has caused widespread damage to the native biodiversity [26, 27]. As a consequence, most of the studies of the natural history of the cane toad derived from introduced populations [28], rather than those in its natural range.

In this study, nine fast-evolving microsatellite loci were used to contrast patterns of genetic variability and population genetic structure along the coast and island populations of *R*. *marina* near the mouth of the Amazon River. Specifically, we aimed to assess the potential role of isolation and genetic drift as mechanisms of differentiation in island populations and deepen the knowledge of the complex interaction of historical and current factors that shape the present-day genetic structure of *R*. *marina*. Finally, we examine levels of malformations and discuss the potential role of island isolation and/or synergetic effects of anthropogenic threats for their occurrence.

## Results

### Genetic diversity

All microsatellites were highly polymorphic across the six populations analyzed, and the number of alleles ranged from 6 (loci 200–12 and RM5) to 19 (RM2) with a mean of 11.8 alleles per locus. No evidence of null alleles, significant allele dropouts or stuttering was identified at the 99% confidence level across all loci. Observations of all pairs of loci in all populations showed no linkage disequilibrium in any case. There was evidence of significant deviations from expected Hardy–Weinberg equilibrium (excess of homozygosity) in four of the nine microsatellite loci (RM2, RM5, RM6 and M200-5), but no locus showed consistent deviations across all populations. A general tendency for an excess of homozygotes was detected in all populations (*F*_*IS*_ >0), although it was statistically significant only for the populations from Marajó Island and Bragança, once the p-value was adjusted for multiple comparisons (Table 1). Standard genetic variability indexes are present in Table 1. Highest genetic diversities (H_O_, H_E_, N_A,_ and A_R_) were found in the largest island population of Marajó, while slightly smaller, but similar values were obtained from the other five populations (Table 1). Private allelic richness (P-AR) was higher in Marajó (0.5) and smaller in Sucuriju (0.30).

**Table 1:** Sample size and standard genetic diversity measures for each sampled locality of *Rhinella marina*.

Population	Locality	N	Na	θ	H_o_	H_e_	AR	P-AR	*F*_*IS*_
**Sucuriju**	Coast	31	6.9	0.536 (0.500–0.576)	0.65	0.73	4.21	0.30	0.11
**Bailique**	Island	20	6.0	0.546 (0.499–0.629)	0.63	0.68	4.07	0.47	0.07
**Marajó**	Island	45	9.6	0.804 (0.746–0.854)	0.73	0.81	4.94	0.51	0.10*
**Algodoal**	Island	29	6.9	0.480 (0.405–0.515)	0.65	0.72	4.09	0.38	0.09
**Bragança**	Coast	22	7.3	0.645 (0.581–0.700)	0.69	0.78	4.63	0.43	0.12*
**Viseu**	Coast	15	6.6	0.936 (0.847–1.120)	0.67	0.76	4.44	0.31	0.12

N, number of individuals; Na, number of alleles observed; θ (population genetic parameter theta, which is equal to four times the effective population size, that is, *N*_*e*_, times the mutation rate, *μ* (4*N*_*e*_*μ*)); H_o_, observed Heterozygosity; H_e_, Heterozygosity within populations; AR, allelic richness; P-AR, private alleles richness; and *F*_*IS*_, inbreeding coefficient.

* significant after Bonferroni correction.

### Population structure and differentiation

**Fig 2:**
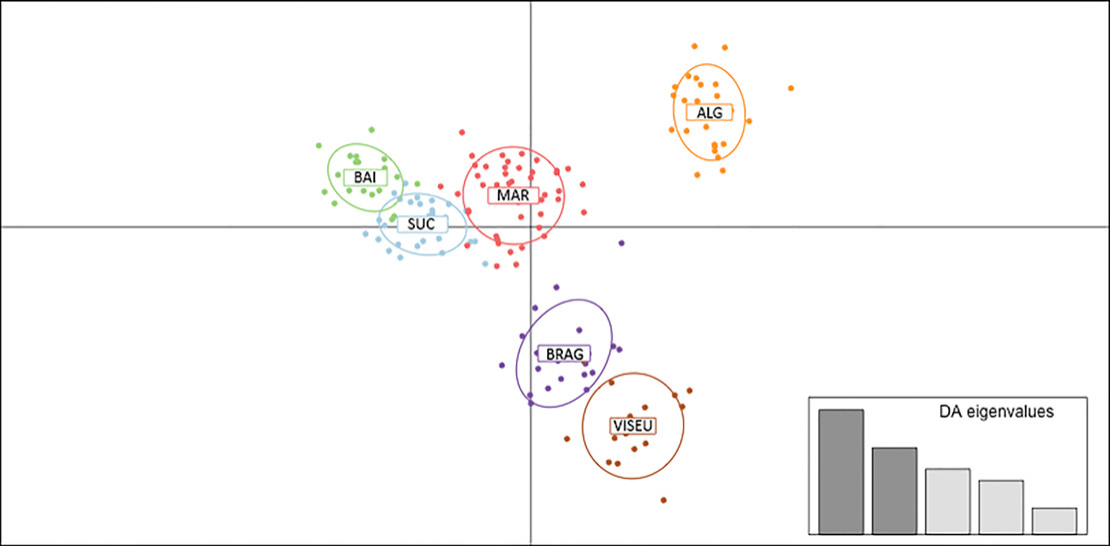
Plot of the first two axes obtained in the DAPC. Color circles represent each individual (the colors represent the same groups presented in Fig 1). The bar graph inset presents the eigenvalues of the five principal components (PCs), in terms of their relative magnitude. BAI, Bailique Island; SUC, Sucuriju; MAR, Marajó Island; ALG, Algodoal Island; BRAG, Bragança; and VISEU, Viseu.

In the STRUCTURE analysis, *K* = 2 was the most optimal solution using the Δ*K* method (Evanno et al. 2005) (S1 Fig). These two main groups correspond to the populations on the right and left margins of the Amazon River. However, based on the distribution of Ln Pr (X/*K*), we found that the likelihood values reached a plateau at *K* values of between 4 and 7 (S1 Fig). The cluster analysis indicated that, for *K* = 6, each sample population corresponds to a distinct cluster, even considering that, in most clusters, a substantial fraction of the ancestry of some individuals is derived from nearby populations (Fig 1). For *K* > 6, new clusters were added to specific populations resulting in a pronounced decrease in the probability of individual assignment to their most probable cluster. Given this, we accepted *K* = 6 as the best representation of the genetic partitioning of our study populations (Fig 1). This level of population structure was entirely consistent with that obtained from the multivariate discriminant analysis (DAPC), which indicated the existence of six groups based on the lowest BIC value (Fig 2; S1 Fig). Interestingly, the first principal component of the DAPC analysis produced a similar pattern of population subdivision to that obtained with STRUCTURE for *K* = 2, grouping the populations according to their respective locations on the right and left margins of the Amazon River (Fig 2). The only exception was the Island of Marajó, which appears to be more closely related with the populations of the left margin. However, the DAPC and STRUCTURE analyses were concordant in showing high levels of admixture among those two groups in this population.

All pairwise *F*_*ST*_ estimates were highly significant, corroborating the differentiation of each population from each other inferred by the STRUCTURE and DAPC analyses (Table 2). The lower level of differentiation was obtained between two closest continental populations (*F*_*ST*_ = 0.048) while the most differentiated populations were the Islands of Bailique and Viseu (*F*_*ST*_ = 0.19).

**Table 2:** Pairwise *F*_*ST*_ values across all sampled populations of *Rhinella marina*.

	Sucuriju	Bailique	Marajó	Algodoal	Bragança
Bailique	0.070475				
Marajó	0.088708	0.105545			
Algodoal	0.151265	0.174402	0.078969		
Bragança	0.130387	0.162143	0.065902	0.088115	
Viseu	0.144568	0.185986	0.099679	0.116404	0.048176

All P-values were highly significant (< 0.001).

### Demography, and contemporary and historical gene flow

Values of theta (ϴ) were similar across all populations, ranging from 0.48 in Algodoal to 0.94 in Viseu (Table 1). Estimates of contemporary migration obtained in BayesAss showed values of gene flow not significantly different from zero across all population comparisons (S1 Table). Historical migration rates (m) estimated by MIGRATE-N also showed values not significantly different from zero across all populations (S1 Table), except for the geographically closest mainland populations Bragança and Viseu (m_Bragança_Viseu_ = 1.458 (1.020–1.784)).

### Deformities

**Fig 3:**
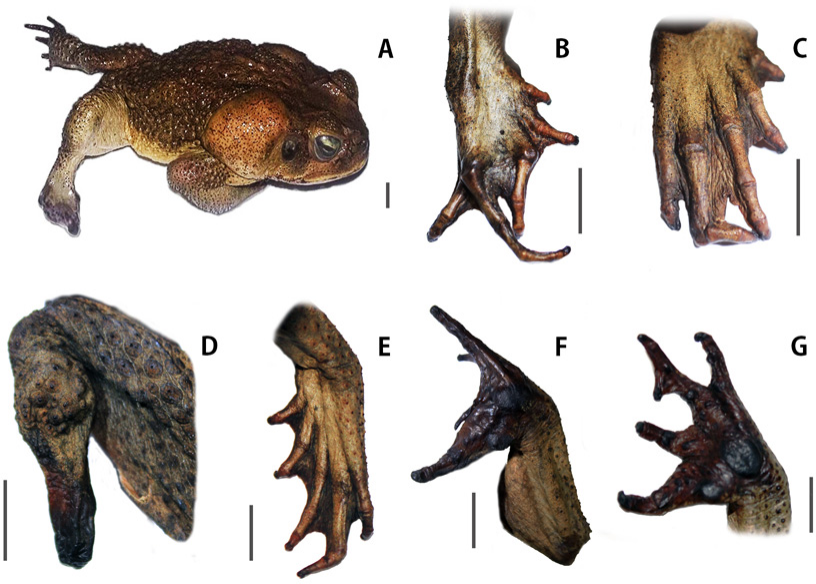
Adult toads (*Rhinella marina*) collected in Bailique Island showing deformities. (A and D) Individuals with Hemimelia (partially missing limb); (B, C and E) Digit bent at joint; (F) Forked digits; Polydactyly (G) (Photographs: Adam R. Bessa-Silva and Davidson Sodré). Scales = 1 cm.

Morphological abnormalities were detected only on Bailique Island. Twenty-six specimens (30%) had one or more deformities, including Hemimelia (partially missing limb), brachydactyly (shortened digits), as well as other malformations of the digits (Fig 3).

## Discussion

### Genetic variability, population structure and differentiation

The cane toad in the Amazon coastal islands system has a complex evolutionary history characterized by substantial population substructure. The Discriminant of Principal Component analyses (DAPC) and the Bayesian multilocus clustering assignment method (STRUCTURE) were congruent in showing that each population was genetically distinct from the others, which are in accordance with the significant *F*_*ST*_ values obtained from all pairwise comparisons. Further, our estimates of both recent and historical gene flow were not significantly different from zero across all population pairs, except the two coastal populations inhabiting continuous habitats (Viseu and Bragança). Given the fundamental pattern of absence/reduced gene flow, the high population differentiation of *R*. *marina* likely reflects the effects of genetic drift resulting from the isolation of island populations. Indeed, geological evidence suggests that those island populations became isolated from the current mainland with the Holocene sea-level rise at least 5,000 to 7,000 years ago [3, 4, 7]. The marine influence, and, therefore, the isolation of coastal areas, started probably much earlier, as evidence of drowning of the Amazon River valley are as old as 11,000 years ago, as the sedimentation at Tapajós River has showed [29]. While several studies have shown that seawater functions as a barrier to the dispersal of amphibians, even over short distances [30–33], it is somewhat intriguing that the populations on Bailique and Marajó Islands, which are fully or partially isolated from the mainland by freshwater substrates, have not experienced migration after their formation.

There is a general assumption that most amphibians have low dispersion capacity due to their physiological constraints [34], and rivers appear to be one of most prominent barriers to gene flow [35]. However, accumulation of empirical data suggests that the barrier effect of rivers in amphibians could not be generalized [36–41]. Although data from introduced *R*. *marina* populations in Australia suggest that this species has a considerable capacity for dispersal [42], studies of natural history in its native range are scarce [43, 44]. Even so, in a recent large-scale biogeographic study of *R*. *marina* [43] based on multilocus analyses, it was found that populations from the north of Amazon river bank were genetically isolated from populations of the opposing south bank, suggesting that Amazon River (with a discharge up to 290,000 m^3^/s) functioned as barrier to dispersal of *R*. *marina*. This hypothesis seems to be supported by the genetic differentiation found here between populations separated by the Amazon River, as revealed by *F*_*ST*_ and STRUCTURE analysis for *K* = 2. Further population substructure found in the species’ natural range on a regional scale also emphasise the importance of historical factors, which are obviously absent in the case of the Australian populations. For example, the occurrence of individuals on the Marajó Island with mixed ancestry derived from the coastal population of Sucuriju (located on the left bank of the Amazon) is likely to be a remnant of historical contacts between the two divergent groups currently separated by the Amazon River.

Many examples of continental island populations investigated to date support the expected pattern of reduced genetic diversity in comparison with mainland populations [32, 33, 45, 46]. Our results, however, suggest that this classic island biogeography prediction is not entirely applicable to our system, since all populations, including both island and the mainland, have similar levels of genetic variability. These results are somewhat surprising given the high levels of population substructure and absence of gene flow, which suggest a relatively long time of isolation under the effects of genetic drift. One possible explanation for this may be associated with the island effective population size since continental islands hold genetic signatures that resulted from vicariance, rather than colonization or founding event as in oceanic islands. Therefore, population size remaining after vicariance will affect the degree of genetic variability loss [32, 47]. Also, the complex geologic history and dynamics of the region would have contributed to the high levels of population substructure and absence of gene flow. As pointed out earlier, the shape of the northern Brazilian coastline has changed significantly during the Holocene, and is currently one of the most extensive and sedimentologically dynamic wetland systems with mangrove vegetation in the world [8, 48]. Indeed, after postglacial sea-level rise all region was under large oscillations of the water discharge and patterns and amounts of sediment deposition and erosion [49], which have contributed to change the geomorphology and by consequence the landscape along the coast [6, 7, 50]. It was reported several changes that have occurred in a short period in the configuration of some islands [50]. This author noted, for example, that sediments gradually filled up the channel that separated the Franco Island from the Porquinhos Island, resulting in the fusion of the two islands. Similarly, the same author reported that the small Veados Island of Araguari River, also due to the filling of the channel, was joined by mainland being part of the right bank of the river. Regarding the Bailique Island, there was an accretion of the coastline near the North channel of more than 10 Km^2^ from 1997 to 2000 [51], as well as for the whole Bailique Archipelago, with an accretion of 82.9 Km^2^ from 1972 to 2008 [52, 53].

Furthermore, the evolution of the Amazonian coast under sea-level rise, during the Holocene as well as at different episodes during the Pleistocene, also changed substantially the ocean tide propagation over the gradually drowned continental shelf. It would result in a gradual increase of the tidal range as demonstrated in other regions [54]. With the drowning of the river valleys and the resulting funnel-like shape of the river mouths, tidal bores (”pororocas”) were certainly frequent and intense in those coastal systems that are called Tidal Rivers [55]. This phenomenon would convert former typical rivers in tidal-dominated, very dynamic environments, which would work as barriers, despite being fresh-water channels. So, considering that the three mainland populations are located close to the sea, two of them on small coastal peninsulas, it is possible that those populations were also isolated from the mainland for long periods. This period of isolation from coastal populations would explain the unexpected high genetic differentiation, absence/restricted of gene flow, and the similar levels of genetic variability with the present-day continental island populations [31]. Interestingly, genetic diversity from all six populations in this study was comparable or even higher to those found in populations of the closely related toad *R*. *schneideri* or other Neotropical amphibians [32, 33], suggesting that those populations of *R*. *marina* seemingly did not suffer a significant reduction in genetic diversity.

A third factor contributing to explain the high degree of population structure and differentiation may be associated with the isolation and genetic differentiation by genetic drift before the formation of the islands [56–58]. While there is evidence supporting the hypothesis of a Pleistocene (prior to island formation) divergence between populations located in opposing margins of the Amazon River [43], population isolation pre- or post-island formation within each of the margins of that river cannot be distinguished correctly. However, considering that the lowest level of differentiation was achieved by the two populations inhabiting continuous habitats (Viseu and Bragança), our data seems to favor genetic differentiation promoted by the absence of gene flow between populations after island formation. Notwithstanding, future analysis of inland populations located relatively far from the current coastline may clarify the effect of the Holocene sea-level changes on patterns of genetic variability in northern Brazilian island populations of *R*. *marina*.

### Conservation implications: malformations and anthropogenic activities

Several studies have already reported the occurrence of phenotypic malformations in *R*. *marina* [59–62]. However, within its native range only on the island of Trinidad were reported abnormalities so far [63]. These authors found that 62% of the examined tadpoles showed some degree of oral abnormalities due to chytrid infection. In the present study, we only detected toads with malformations in the island of Bailique, where 30% of all examined specimens exhibited one or more abnormalities (Fig 3). Considering that the frequency of abnormalities in natural populations of amphibians is usually less than 5%-10% [64–66], the frequency of individuals with malformations in Bailique Island should be regarded as intriguingly high and of utmost importance in a conservation point of view.

Malformations in natural populations can be an important indicator of inbreeding [67] or stress during the development stability of organisms [68–70]. High levels of inbreeding may increase the risk of infertility, and reduced reproduction rates and survival, thus exposing these populations to stochastic factors such as the fixation of deleterious alleles [71]. Here, we found a general tendency for an excess of homozygotes in all populations, as shown by the positive *F*_*IS*_ values. However, significant *F*_*IS*_ values were only found in the Marajó Island and Bragança populations, which not presented any evidence of malformations. In amphibians, one of the world’s most threatened vertebrates group, malformations are mainly associated with abiotic (*e*.*g*., UV radiation and different physicochemical stressors) and biotic factors (*e*.*g*., predation and pathogen infections) or by a synergistically interactions of these factors [68, 70]. All the study area, including islands, is under pressure from a broad range of anthropogenic activities, industries, farming and ranching, which have contributed to landscape changes and fragmentation of natural habitats. A major impact on both Marajó and Bailique Islands is the domesticated water buffalo ranching, which is associated with the disappearance of native vegetation, pollution, increased deposition of sediments in freshwater environments and the replacement of native vegetation by the highly invasive toxic plant, *Ipomea fistulosa* [72]. Furthermore, the region of Bailique Island, with a rich hydrographic network, mainly linked with the Araguari River [22, 73], has been increasingly affected by several other anthropogenic activities, including the building of artificial channels, hydroelectric plants, and in particular, the exploitation of natural mineral resources. Still, some studies suggested that mining activity, located mainly in the Serra do Navio region, adjacent to the Araguari River, has contributed to the accumulation of ^210^Pb and high mercury concentrations [19, 22] on the Araguari River. Accordingly, it is possible that the high percentage of individuals with malformations found in Bailique is associated with the occurrence of freshwater contaminants. Indeed, several studies combining field and laboratory investigations have suggested that environmental contaminants in pond sediments of breeding sites (heavy metals and petroleum hydrocarbons) are responsible for the abnormalities observed in some anuran species [64, 74], including the cane toad in Bermuda's island [60–62]. In this Island, approximately 30% of adults and 24% of metamorphs presented malformations, including ectromelia, ectrodactyly, missing or misplaced eyes, spinal and pelvic abnormalities, and a variety of limb malformations. Despite the shreds of evidence of the presence of chemical stressors on the Island of Bailique, further investigation of other potential causes is required.

### Conclusion

The cane toad is one of the world’s most successful invader species, and is also one of the most intensively studied invasive taxa [28]. However, the relative abundance of information on the natural history of this species has mostly been collected in exotic areas, whereas few data are available from its natural range [43]. Indeed, our research represents one of the few studies investigating the population genetic structure of the cane toad in its natural range. In the present study, we provided evidences that current substructure of the cane toad populations may represent remnants of an ancient, more or less continuous habitat that was fragmented into smaller, isolated areas during to the rise in sea level in the Holocene. This network of islands system is comparable to habitat fragments in a human-modified landscape, so the observed patterns of genetic structure in the cane toad populations may provide insights into the potential effects of the isolation populations in other regions subject to similar patterns of habitat disturbance. This is of particular interest for future conservation planning, given that the expansion of the beef and soybean industries in the Amazon basin has led to an increase in deforestation rates. One other intriguing finding of our study was the high percentage of individuals with abnormalities found on Bailique Island. Although further investigations will be needed to determine the exact causes of this phenomenon, the a more effective control of human activities, especially those related to the production of chemical stressors and the discharge of contaminants into the water will be crucial to the development of a comprehensive conservation strategy.

Finally, considering the genetic uniqueness of each cane toad population together with their high natural vulnerability and the intense human pressures affecting of this complex system of continental islands, we recommend that these populations should be treated as discrete units for conservation management purposes.

## Material and Methods

### Ethics statement

The samples analyzed in the present study were obtained in accordance by the requirements of Brazilian environmental legislation, being approved by the federal Chico Mendes Institute for Biodiversity Conservation (ICMBio), through license number 38047–3. The individuals were euthanized (according to the Brazilian legislation, law 11,974, being authorized by the Ethics Committee of ICMBio followed by Universidade Federal do Pará) using an anesthetic application (5% Lidocaine) over the skin to minimize animal suffering, as recommendations of the Herpetological Animal Care and Use Committee (HACC) of the American Society of Ichthyologists and Herpetologists. The Marajó Island population (city of Soure) is located in the Marajó Archipelago Environmental Protection Area, and the collection and transportation of specimens from this reserve was authorized by the same license, which also covered the collection and transportation of specimens from the other sites, located on privately-owned or unprotected public lands.

### Study area and sampling

Cane toad samples were collected from populations of three mainland areas (Sucuriju, Bragança and Viseu) and three continental islands (Bailique, Algodoal and Marajó) (Fig 1, Table 1). The Bailique and Marajó Islands are both located at the mouth of the Amazon River, primarily isolated by freshwater environments. The water body surrounding Bailique Island (230 km^2^) is relatively uniform, and its distance from the mainland is approximately 2 km. The Marajó Island, with an area of approximately 40,100 km^2^, is the biggest island of the region, and it is isolated from the continent by long distances on its seaward side (76 km). However, its riverward side (Amazon estuary) is separated from the mainland by only short distances (0.64 km), through a complex network of tidal channels, creeks and other smaller water bodies. The Algodoal Island (19 km^2^) is located on the continental shelf and is isolated from the mainland of only *ca*. 0.20 km. In contrast with both Bailique and Marajó Islands, Algodoal is essentially under the influence of the marine environment. Maps were created in ArcGIS 10.2 ESRI (Environmental Systems Research Institute).

In small and isolated populations, such as those of islands, genetic drift can lead to a random loss of alleles with consequent survival and fitness reductions and appearance of malformations. Accordingly, we performed a detailed external morphological examination of all specimens both in the field and in the laboratory, using both photographs and a magnifying glass to investigate the presence of malformations. The number of individuals analyzed per population was similar for both morphological and genetic analysis, except Bailique Island, where only 20 of 88 collected toads were genetically characterized. Tissue samples (obtained from the abductor muscle) were preserved in 92% ethanol for subsequent extraction of DNA.

### DNA extraction and genotyping

Total genomic DNA was isolated using a PROMEGA extraction kit following manufacturer’s protocol and diluted to a final concentration of 40 ng/μl. Nine microsatellite loci were amplified with polymerase chain reaction (PCR), following the conditions described by [75]. Microsatellite genotyping was performed with fluorescently labelled primers, in an automated sequencer (ABI 3500xl sequencer; Applied Biosystems) using an internal size standard (Rox– 500; Applied Biosystems), and analyzed in GeneMapper 3.7 (Applied Biosystems), as described by above.

### Data analysis

Micro-Checker 2.2.3 software [76] was used to detect null alleles, dropout and stuttering, while Arlequin 3.5 [77] and Genodive 2.0b27 [78] were used to investigate significant deviations from Hardy-Weinberg equilibrium and linkage disequilibrium, based on the Markov chain approach, with 10,000 permutations and significance of α = 0.05. A Bonferroni correction for multiple analyzes was subsequently conducted [79].

Standard genetic variability measures, including observed (Ho) and expected (He) heterozygosity, and the mean number of alleles (Na) were obtained in Genodive 2.0b27. The allelic richness and private alleles richness were estimated in HP-Rare [80], which uses the rarefaction of alleles and hierarchical sampling to adjust and minimize the effects of unequal sample size across populations.

### Population structure

Genetic differentiation among populations was estimated as *F*_*ST*_ [81], using Arlequin 3.11. We used the Bayesian clustering assignment multilocus approach implemented in STRUCTURE 2.3.4 to infer population structure [82]. We first performed, under the admixture ancestry model, 20 independent runs for each *K* ranging from 1 to 10, using 10^7^ MCMC (Monte Carlo Markov Chain) repetitions and discarding the first 5x10^5^ iterations as burn-in. The most probable value of *K* (clusters) that captures the most population structure in the data was identified monitoring the estimated log posterior probability of the data (ln Pr (X/*K*) [82], and estimating the second-order rate of change of the likelihood function (Δ*K*) [83]. To assist in the selection of the most probable value of *K*, post processing STRUCTURE runs were done by the software STRUCTURE Harvester v0.6.93 [84]. This program produces an output that includes graphical files representing ln Pr (X/*K*) and Δ*K* of [83] per *K* and repeated run. The Clumpp 1.1.2 program [85] was used to align the membership coefficients from the 20 replicates for each *K*-value based on the LargeKGreedy algorithm. Graphical representations were performed in Distruct 1.1.2 [86].

The analysis of population structure was also based on a multivariate approach, DAPC (Discriminant Analysis of Principal Components), implemented in Adegenet package [87] for R 2.15.1. This method uses the Bayesian Information Criterion (BIC) to identify the most probable value of *K* (varying from 1 to 14), based on the relationship between the number of clusters (*K*) and the smallest BIC value.

Estimates of recent migrations between populations were obtained in the BayesAss 1.3 software [88]. This Bayesian approach is based on a MCMC and does not require the data to be in Hardy–Weinberg equilibrium [88]. Initial testing indicated that convergence is reached after 3 x 10^6^ iterations of the MCMC and delta values of 0.15 for *p* (allele frequency), *m* (migration rate), and *f* (inbreeding), which generated acceptance rates within the limits recommended by the authors (40–60%). The final analyses were based on 3 x 10^7^ MCMC iterations, sampling at every 2000^th^ increment, and 1 x 10^7^ of burn-in. Long-term migration rates and the population genetic parameter theta (θ) were investigated using Migrate-N [89, 90], which uses coalescence and MCMC to estimate historic gene flow, or M (M = m/μ, where m = migration rate per generation, and μ = mutation rate). For this analysis, a Brownian approximation model (which is similar to the stepwise mutation model) was applied in a Maximum-Likelihood approach, with a constant mutation rate for all loci. The estimates of *F*_*ST*_ and an UPGMA tree were used as the initial parameters for this analysis, with an island model being used to determine the migration rate between populations. The estimates of M were obtained based on ten short chains and five long chains with 10,000 and 100,000 recorded genealogies, respectively, and burn-in of 10,000 for each chain. The analyses were run three times to evaluate the consistency of the results.

## Supporting Information

S1 Fig Bayesian clustering results. Results of STRUCTURE analysis for microsatellites (*K* = 2–6) (A); best number of clusters (L(*K*)) according to STRUCTURE (B); the highest ln-probability and the Δ*K* optimality criteria (C); and results of BIC (D).
(TIF)

S1 File Microsatellite data (Genepop format). (TXT)

S1 Table Asymmetrical migration rates estimated by MIGRATE-N (A) and BayesAss (B). The values are presented by the means and 95% CI of posterior distributions. Bold means significant values. The values in the diagonal of B are the proportion of non-immigrants samples in each generation.
(DOCX)
