# Supplementary figures and images for: Patterns of Genetic Variability in Island Populations of the Cane Toad (Rhinella marina) from the Mouth of the Amazon
Source: PLoS One. 2016 Apr 13;11(4):e0152492. doi: 10.1371/journal.pone.0152492 (PMC4830453; doi:10.1371/journal.pone.0152492)

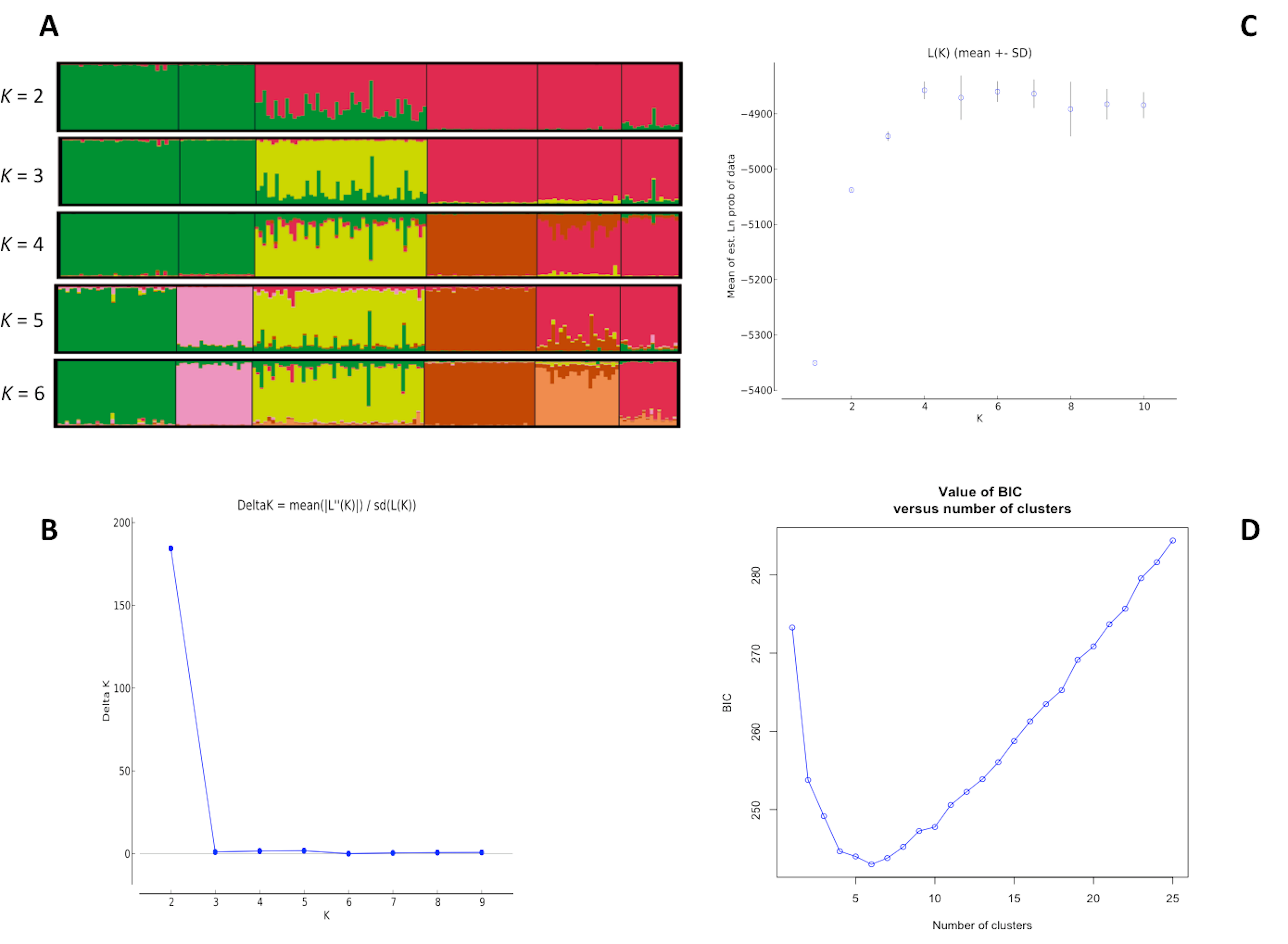

Supplement: S1 Fig — Results of STRUCTURE analysis for microsatellites (K = 2–6) (A); best number of clusters (L(K)) according to STRUCTURE (B); the highest ln-probability and the ΔK optimality criteria (C); and results of BIC (D). (TIF) [file pone.0152492.s001.tif]
