# Supplementary material for: Patterns of Genetic Variability in Island Populations of the Cane Toad (Rhinella marina) from the Mouth of the Amazon
Source: PLoS One. 2016 Apr 13;11(4):e0152492. doi: 10.1371/journal.pone.0152492 (PMC4830453; doi:10.1371/journal.pone.0152492)
Supplement: S1 Table — The values are presented by the means and 95% CI of posterior distributions. Bold means significant values. The values in the diagonal of B are the proportion of non-immigrants samples in each generation. (DOCX) [file pone.0152492.s003.docx]

| A |  |  |  |  |  |  |
| --- | --- | --- | --- | --- | --- | --- |
| M | Sucuriju | Bailique | Marajó | Algodoal | Bragança | Viseu |
| Sucuriju | - | 1.199(0-3.959) | 1.086(0-2.484) | 1.071(0-4.560) | 0.894(0-2.384 | 1.080(0-4.452) |
| Bailique | 0.501(0-1.442) | - | 0.846(0-2.610) | 1.023(0-2.983) | 1.027(0-2.105) | 1.055(0-2.133) |
| Marajó | 0.980(0-3.763) | 0.779(0-3.014) | - | 1.420(0-5.144) | 0.829(0-2.299) | 0.995(0-3.739) |
| Algodoal | 0.983(0-2.061) | 1.000(0-2.940) | 0.879(0-3.701) | - | 0.941 (0-2.195) | 1.227(0-3.627) |
| Bragança | 1.378(0-3.401) | 0.760(0-2.502) | 1.099(0-2.659) | 0.953(0-2.205) | - | 1.129(0.861-1.440) |
| Viseu | 0.758(0-1.813) | 0.7638(0-1.778) | 0.774(0-2.219) | 0.787 9(0-1.759) | **1.458 (1.020-1.784)** | - |
| B |  |  |  |  |  |  |
| Source | Sucuriju | Bailique | Marajó | Algodoal | Bragança | Viseu |
| Sucuriju | 0.980(0.941-0.999) | 0.005(-0.011- 0.022) | 0.004(-0.010-0.020) | 0.003(-0.008-0.014) | 0.03(-0.007-0.013) | 0.003(-0.007-0.014) |
| Bailique | 0.005(-0.014-0.025) | 0.980(0.930-0.999) | 0.003(-0.010-0.017) | 0.003(-0.010-0.017) | 0.003 -0.009-0.016) | 0.003(-0.009-0.016) |
| Marajó | 0.012(-0.010-0.035) | 0.005(-0.010-0.022) | 0.960(0.903. 0.993) | 0.005(-0.009-0.019) | 0.009(-0.014-0.032) | 0.007(-0.011-0.026) |
| Algodoal | 0.002(-0.007-0.012) | 0.002(-0.007-0.012) | 0.002(-0.007-0.012) | 0.986(0.951-0.999) | 0.003(-0.008-0.014) | 0.002(-0.007-0.013) |
| Bragança | 0.004(-0.010-0.019) | 0.004(-0.009-0.018) | 0.006(-0.013-0.025) | 0.021(-0.024-0.066) | 0.956(0.881-0.998) | 0.006(-0.015-0.029) |
| Viseu | 0.007 -0.016-0.030) | 0.006(-0.015- 0.028) | 0.007(-0.017- 0.033) | 0.007(-0.016-0.030) | 0.025(-0.028-0.080) | 0.945(0.860-0996) |

Table S1- Asymmetrical migration rates estimated by MIGRATE-N (A) and BayesAss (B). The values are presented by the means and 95% CI of posterior distributions. Bold means significant values. The values in the diagonal of B are the proportion of non-immigrants samples in each generation.
